# Parent’s Stress Predictors during a Child’s Hospitalization

**DOI:** 10.3390/ijerph182212019

**Published:** 2021-11-16

**Authors:** Agata Zdun-Ryżewska, Natalia Nadrowska, Magdalena Błażek, Katarzyna Białek, Ewa Zach, Dagmara Krywda-Rybska

**Affiliations:** 1 Department of Quality of Life Research, Faculty of Health Sciencies, Medical University of Gdansk, 80-210 Gdansk, Poland; natalia.nadrowska@gumed.edu.pl (N.N.); blamag@gumed.edu.pl (M.B.); 2 Szpital Dziecięcy Polanki im. Macieja Płażyńskiego w Gdańsku sp. z o.o., 80-308 Gdansk, Poland; kasiajoasiab@gmail.com (K.B.); evazach@wp.pl (E.Z.); psycholog-polanki@wp.pl (D.K.-R.)

**Keywords:** psychosocial diagnosis, environmental and family conditions, psychological assessment, stress, caregiver, parent, hospitalization

## Abstract

A child’s illness and hospitalization are particularly difficult and most often an unpredictable situation in a family’s life cycle. The level of stress of a parent of a hospitalized child depends on many factors, such as the psychological characteristics of the child and the parent, the child’s health condition, and support from the family and medical staff. Our research aimed to search for interactions between the stress experienced by the parent and the temperamental variables of both the child and the parent, and the support received from the family and hospital staff. Using three pencil-paper questionnaires—PSS, EAS-D, EAS-C—and interview questionnaire, we tested 203 parent–child dyads at the time of children hospitalization. It was revealed that the most notable moderator of the relationship between temperamental traits and the characteristics of the hospital-related situation is the child’s age. When analyzing the situation of a family with a hospitalized child, particular attention should be paid to parental emotional distress, which, regardless of the child’s age, predicts a high level of parental stress.

## 1. Introduction

The hospitalization of a child is a highly stressful experience for both the parents and their child. An important role during the child’s stay in hospital is played by the support of the medical staff and the psychological characteristics of the child and the parents, as well as the relationship between them. Particularly important predictors of the well-being of the parents and their child in this difficult situation for the family include the quality of communication concerning the first factor (staff support), mainly in the area of feedback and the possibility of participating in childcare [1,2], and the characteristics of styles of coping with stress, emotionality, and temperamental conditions concerning the second factor. Many parents experience high levels of stress (anxiety, depression, insecurity) when their child is hospitalized [3], and research in this area often focus on specific groups of children, such as children with cancer [4,5], infants with low birth weight [6], or specific hospital experiences such as intensive care unit stay [7] or cardiac surgery [8]. As shown by research studies, strong parental anxiety related to the child’s hospitalization may lead to a deterioration in the functioning of the whole family, whose function is largely dependent on the child’s stay in the hospital [9,10].

The analysis of the way parents and children react to their stay in a hospital requires taking into account not only personal factors, such as temperamental conditions or styles of coping with stress but also the perspective of the family system. It is not without reason that the expression ‘when a child is ill, the whole family becomes ill’ exists. Therefore, we can identify three types of determinants: the individual characteristics of the child, the individual characteristics of the parents, and the interaction of these factors from the perspective of mutual relations. In 1994, Bonn [11] reviewed research on the psychological responses of children to hospitalization. The results of this review revealed that regardless of individual differences in stress resistance and different ways of coping with it, children experience emotional problems during hospitalization, especially when it is prolonged and repeated. The impact of illness and hospitalization can be so significant that it affects even the child’s development, causing significant delays. One of the very interesting research papers involving children with leukemia showed an average of three-month delays in all developmental areas studied (with the greatest delays in the area of socialization, motor, and daily living skills) one year after treatment. More days of hospitalization, in combination with a child’s negative emotionality, creates an evident risk for developmental delays [12].

In addition to the child’s age, individual factors such as temperament and intelligence influence the coping style and thus the short-term as well as long-term effects of hospitalization. Studies have shown that children who use active coping strategies cooperate better with hospital staff and show fewer emotional problems, both during and after hospitalization.

While the importance of the child’s age, as well as the number of hospital stays, was analyzed relatively often, the importance of temperamental traits as predictors of adaptation, emotional reactions, and functioning in hospitalization is very rare. The study by Lumley, Melamed, and Abeles (1993) was one of the few, in which temperamental traits were treated as predictors of the child’s response to anesthesia, and the influence of the interaction between the child’s temperament and mother’s behavior on this response was analyzed [13]. The results showed that children with a less adaptive configuration of temperamental traits (approach and withdrawal dimension), i.e., more withdrawn and less adaptable to new stimuli, were more stressed.

On the other hand, the study by Bonn (1994), in which mothers were asked to assess the temperament of their children, revealed that those who were assessed by mothers as moodier, less flexible, and more withdrawn were less able to cope with hospitalization [11]. There is a lack of studies that would take into account the individual characteristics of the child, but also those of the parents, and the effects of interactions between these characteristics, the style of reaction, and emotionality in the situation of a child’s hospitalization. Little is known about the psychological functioning of children/parents/families in the situation of a child’s stay in a general pediatric ward. However, clinical observations clearly indicate that the high level of parental stress during the child’s hospitalization is an obstacle to effective involvement in childcare, may negatively affect the child and is associated with adaptation difficulties of a child, and may also affect the recovery process. High levels of parental stress, anxiety, insecurity, and a negative coping style contribute to the emergence of long-term emotional effects, such as anxiety, depression, and even the symptoms of post-traumatic stress disorder. The study conducted by Melnyk and Feinstein (2001) [14] on the group of 49 mothers of young children (age 24–68 months) indicates that negative behavior of a child was mediated by maternal anxiety and the possibility to participate in the care during hospitalization. As the authors point out, it is very important to inform and involve mothers in order to lower the distress experienced by a child. Kirkby and Whelan (1996) point out that negative emotional reactions of a parent strongly influence a child’s ability to cope [15]. We can conclude that when a parent’s anxiety is high, and he/she experiences emotional distress, the child is more likely to become less cooperative and more anxious.

An interesting study exploring the interaction of a child’s temperamental characteristics, the child’s parent, and behavioral problems in the child [16] showed that the goodness-of-fit hypothesis formulated by Chess and Thomas (1991) [17]—i.e., if the environment does not provide a good fit, behavioral symptoms that are temperamental traits of difficult children are likely to develop into more serious problems, leading to behavioral disturbances—is applicable when a child is hospitalized. The smaller the fit is, the more likely are the strong negative emotional reactions to the hospitalization situation, and the less adaptation a child shows towards those reactions.

The importance of the reactions of parents and their child to the hospital stay, the interaction between them, and the support of healthcare professionals become especially powerful when we realize that all these factors are directly related to the recovery process and may either quicken or delay it. In this article, therefore, we explore the complex relationships of the temperamental characteristics of the parents and their child, their reaction to stress, and staff support, highlighting their role in determining the course of hospitalization, its short and long-term consequences, and impact on the treatment process.

## 2. Materials and Methods

### 2.1. Aims of the Study

Our main goal was to examine the interaction of the temperamental traits of the child and the parent in a stressful situation when the child is hospitalized. First, we searched for relationships between the child’s health subjectively perceived by parents, disruption of life by the child’s illness, the support obtained, and the intensity of parental stress. Secondly, we decided to check the moderating role of the child’s age on the relationship between subjective assessment of the child’s health by the parent, received support and disruption of the routine of family life, the temperamental traits of the child and the parent, and the stress experienced by the parent.

### 2.2. Procedure and Participants

We obtained approval for our project from the Independent Bioethics Committee for Scientific Research at the Medical University of Gdańsk (protocol code NKBBN/93/2016 and date of approval: 17 May 2016). The study participants were 203 parent–child dyads. These were recruited from among children hospitalized at the Polanki Children’s Hospital in Gdańsk, Poland, at the time of our study.

The parents participating in the study were 177 (87.2%) female and 26 (12.8%) male, and their mean age was 35.65 (SD = 5.48). Most caregivers were married (*n* = 159, 78.3%) with higher education (*n* = 115, 56.7%), employed under a contract of employment (*n* = 139, 68.5%) with a good level of income (*n* = 97, 47.8%). The main person taking care of the child in the hospital were mothers (*n* = 146, 71.9%), joint care was reported by 40 respondents (19.7%), and the father was the main caregiver only in 6.9% (*n* = 14). Charactersitcs of parent’s group are presented in Table 1.

The age of the hospitalized children ranged from 3 to 14 years (M = 6.75, SD = 2.32). There were 109 girls (53.7%) and 94 boys (46.3%). Children who did not suffer from chronic diseases participated in the study. Most of the studied children suffered from upper respiratory tract infections, pneumonia, and bronchitis. The length of the children’s hospitalization ranged from 1 day to 23 days (M = 3.44, SD = 2.77). Some of the patients were hospitalized once (*n* = 59, 29.1%), but most (*n* = 144, 70.9%) were repeatedly hospitalized (2 to 5 times). All data are gathered in Table 2.

### 2.3. Measures

Parents participating in the study completed an interview questionnaire and the following methods: Perceived Stress Scale and The EAS Temperament Survey in two versions: EAS-TS for Adults (EAS-D) and EAS-TS for Children: Parental Ratings (EAS-C).

The interview questionnaire contained questions about elementary demographic data, such as gender, age of the participants, education, marital status, employment, and income of the parents. The questions also concerned the situation of the child’s illness in which the family found themselves, such as childcare in the hospital, duration of stay, or the number of hospitalizations. We also asked four additional questions with a subjective assessment: the child’s health, disruptions to family life caused by the child’s illness, and perceived support from family and hospital staff. The respondents assessed these items on a 10-point scale (from 1 to 10), where 1 indicated the child’s very poor health, lack of support, and no life disruptions, while 10 indicated very good health of the child, high level of support, and a serious crisis in family life.

The Perceived Stress Scale [18], adapted by Juczyński and Ogińska-Bulik (2012) [19], was used to measure the intensity of stress related to one’s own life situation. It is a 10-item scale on which the respondents rate the perceived stress from 0 (never) to 4 (very often). The higher the score on the scale of perceived stress is, the lower is the psychological comfort associated with a difficult situation. Cronbach’s alpha for this method is 0.86, and it is recommended for the diagnosis of healthy and afflicted people.

To measure temperamental traits, we used The Temperament EAS Survey [20] adapted by Oniszczenko (1997) [21], in two versions: EAS-TS for Adults (EAS-D) and EAS-TS for Children: Parental Ratings (EAS-C). Each of these versions consists of 20 items, which are assessed by the respondents on a 5-point scale (from 1—definitely not to 5—definitely yes). The EAS-D version consists of five scales that characterize the temperament, such as emotionality—distress, emotionality—anger, emotionality—fear, activity, and sociability. The EAS-C contains the following four scales: shyness, sociability, activity, and emotionality. Both versions of the questionnaire have satisfactory psychometric properties (Oniszczenko, 1997) and can be used in individual and group research.

### 2.4. Statistical Analyses

IBM SPSS Statistics 24 was used to perform statistical analyses. We used Pearson’s r correlation coefficient to assess the strength and direction of relationships between variables, and we performed a multinomial regression analysis with interaction effect to test the moderating role of the child’s age. The moderation analysis consisted of three steps [22]. The first was to introduce parental stress predictors to the model of regression. The second was based on the interaction between predictors and age. When the interaction between the stress predictors and age was found to be significant, we proceeded to the third step, which involved carrying out a regression analysis divided into two groups: younger children (3–6 years old) and older children (7–14 years old). The division criterion of age was established based on Erikson’s theory of psychosocial development [23].

## 3. Results

### 3.1. Correlations between Study Variables

Pearson’s r correlation analysis revealed moderate and positive correlations between the severity of parental stress and their emotionality—distress (r = −0.55, *p* < 0.001) and emotionality—anger (r = 0.38, *p* < 0.001). Moreover, we observed that with an increase in a child’s emotionality (r = 0.28, *p* < 0.001) and the fear of the parent (r = 0.22, *p* < 0.01), the parent’s stress also increased. We also observed that high parental stress is correlated with low support from relatives (r = −0.19, *p* < 0.01) and the deteriorating health of the child (r = −0.16, *p* < 0.05). These correlations are weak, which should be taken into account when interpreting the results. Table 3 and Table 4 show these correlations.

### 3.2. Moderation Analysis

The moderation analysis was carried out in three steps, and we also assumed the child’s age has an effect on the interaction between the variables included in the model. In the first step, predictors were introduced into the model, which explained 34.3% of the variance in the intensity of perceived parental stress. Significant predictors were revealed to be parental emotionality—distress (β = 0.54, *p* < 0.001) and parental activity (β = −0.14, *p* < 0.001).

In the second step of the analysis, we introduced the interaction component in the form of age into the model. The model takes into account the interaction effects explaining 36.8% of the variance in stress parents. In this model, we found one significant predictor, parental emotionality—distress (β = 0.56, *p* < 0.001), and two significant effects of interaction between the child’s age and the parent’s assessment of the child’s health (β = 0.14, *p* < 0.05) and the child’s sociability (β = −0.17, *p* < 0.05). The first and second steps of moderation analysis are presented in Table 5.

Obtaining statistically significant effects of the interaction between the child’s age and independent variables allowed us to conduct separate multiple regression analyses in two groups: younger children (3–6 years old) and older children (7–14 years old). The predictors introduced into the model explained 41% of the variance in parental stress in the group of younger children. The severity of the caregiver’s stress was predicted by the parental emotionality—distress (β = 0.62, *p* < 0.001) and emotionality of the child (β = 0.24, *p* < 0.05). On the other hand, in the regression model in the group of older children, we observed the following significant predictors: the child’s health status as perceived by the parent (β = −0.19, *p* < 0.05) and parental emotionality—distress (β = 0.49, *p* < 0.001). Moreover, this model explains more than 50% of the variance in parental stress. Table 6 and Table 7 present the regression analyses by group according to the age of the child.

Thus, we obtained results confirming the moderating role of the child’s age in the relationship between temperamental and health variables and parental stress. In the group of parents of older children, with the increase in parental emotionality—distress and lowering of the child’s health assessment, psychological discomfort increases. In contrast, in the group of parents of younger children, parental stress increases with the increase in parental emotionality—distress and child emotionality.

## 4. Discussion

Parents’ stress during their child’s hospitalization is an integral part of this experience, which is confirmed by many studies [24,25]. Most of them focus on the stress experienced by parents of a chronically or very seriously ill child, but the need for a deeper analysis of stress also in short-term hospitalizations is emphasized more and more often [26]. Parents transitioning from a regular caregiver to a child’s inpatient caregiver might experience severe stress and anxiety over the child’s health, which is completely understandable if it deteriorates to the point that the child requires hospitalization. This sometimes unforeseen and sudden change is connected with laying aside current personal commitments and obligations, as well as difficulties in performing family, professional, and social roles. This is very often followed by deprivation of physical and emotional needs combined with growing fatigue. The stress increases with the increasing number of hospitalizations and an increase in their duration [1].

The above-mentioned reasons underline why it is worthwhile to diagnose the stress experienced by a parent in the situation of a child’s hospitalization. Understanding this phenomenon can bring us closer to an effective intervention aimed at helping the entire family system.

Our results allowed us to predict parental stress and confirmed the moderating role of the child’s age in this process. In the literature, we can find contradictory reports as to whether the child’s age is relevant to the parent’s stress in case of illness. According to some sources, there is no difference in the intensity of stress experienced by parents of younger and older children [27]. Other sources indicate the similarity of the needs of the parents of hospitalized children, which are also independent of the child’s age, with the main need observable in all parents, which is the need for restoring security [28]. Our earlier research showed some connections between the age of the child and the parent’s stress. Parents of younger children were found to be more stressed [29]. In the current research, without resolving this dilemma, we were able to identify other predictors of stress in parents of younger and older children. Even with a similar level of parental stress in both groups, other variables seem to be important for understanding its phenomenon. In the group of parents of older children, their distress increases with the increase in parental emotionality and with the worsening of the child’s health condition. In the group of parents of younger children, with the increase in parental and child’s emotionality, psychological discomfort increases.

The connection between a parent’s stress and the health condition of a child is obvious and well documented. Having a child with any health problems immediately creates a risk for an elevated level of stress for a caregiver [30,31,32]. It would seem that the stress of parents of older children, which is directly related to the assessment of the child’s health, is more “logical” (in a sense, easier to understand) than the stress of parents of younger children. A child’s health status is not a significant predictor of stress for parents of younger children, which seems surprising at first. There may be several explanations for the obtained result. With age, the emotional and cognitive development of a child progresses. It can be assumed with a high degree of probability that the older the child, the greater his/her ability to comprehend psychological and somatic conditions, and the greater communication skills, and emerging stress coping skills, which, if constructive, may support the process of adaptation to hospital conditions. The age of a child, usually directly related to the stage of mental development, is of great importance for the adequate image of the disease and his/her adaptation. In addition, with younger children, we are often dealing with lower awareness of the disease and the use of defense strategies rather than task strategies due to the greater emotionality and passivity when compared with older children [33]. All of this can affect the type of interaction parents have with their ill children in the hospital. In the case of older children, their subjectively perceived health may have a greater and more direct impact on the parent’s stress. In the case of younger children who cannot communicate well, the parent may rely more on the emotions and child’s behavior (tearfulness, tension, motor restlessness, or changes in existing patterns of behavior), and this is more important for the stress experienced.

An additional explanation why the emotionality of a younger (not as much as an older) child is a predictor of parental stress could be a result of the unquestionable fact that small children in hospitals are much more difficult to care for. The risk of emotional difficulties is positively related to age. Younger children may have additional difficulties adjusting to hospital conditions due to the high need for activity, which will be highly frustrating due to the limitations in movement in the hospital area and the often health-related injunction to stop strenuous physical activity [33].

This brings us to, in our opinion, the most important result obtained in this research. In other studies, especially in the medical field, when describing parental stress, attention is focused on various variables easy to grasp from the perspective of a doctor or nurse, such as the child’s health, being single or married, gender, or sources of social support. In our research, in the case of parental stress (regardless of the age of the child), its most important predictor was the temperamental variable in the form of parental emotionality—distress. This is one of the dimensions from the multidimensional and causal personality model described by Buss and Plomin [20]. It refers to the quality and intensity of emotional reactions. High emotionality is connected with reacting even to low-intensity stimuli with negative emotions such as sadness or anxiety, which are difficult to control. Difficulties in maintaining calmness, combined with high sensitivity to stimuli that cause dissatisfaction, are also typical for high emotionality. On the contrary, low emotionality is associated with emotional stability, which is at the other end of this dimension. This construct is close to the combined concepts of neuroticism and harm avoidance.

When defining a child’s hospitalization as a parent’s stressor we can observe it from a transactional model perspective. Stress, as a condition connected with perceiving the discrepancy between the demands of the situation and the parent’s resources to cope with those demands, requires adaptation [34]. High sensitivity to stimuli with quick and uncontrollable emotions as a response to adverse events probably influences primary and secondary appraisal and explains the stronger stress reactions. It also affects coping with stress and adapting to the hospitalization process. From the family system’s perspective during hospitalization, there are three major tasks: to handle emotional challenges, establish a relationship with the medical team to ensure effective collaboration, and manage changes/conflicts inside the family [35]. Handling emotional challenges might be particularly difficult for parents with high emotional distress, which potentially might influence the management of changes and effective collaboration.

It is puzzling why the level of support, especially from medical staff, with whom good cooperation is of such great importance for the child’s treatment process, does not help predict the parent’s stress level. Many sources indicate the importance of supporting medical personnel for the stress of the patient and their family [36,37,38]. Our results do not support these reports. Social support is extremely important, but it may be related to variables that were not included in this study. We see the need for further research on this topic. It seems that this temperamental dimension is the most important in the diagnosis aimed at assessing the psychological condition (especially level of stress) of the parents of the hospitalized child.

In one research paper, most parents that constituted the study group viewed accompanying their hospitalized child as an unconditional aspect of being a parent what was connected with a strong desire for participation [36]. “In our child-oriented culture, we tend to stress the obligations of the parents and pay less attention to their rights” [39]. Focusing on parental stress might help the whole family system. Our results are in line with family system frameworks, with their broad and multidisciplinary models and one commonly shared view of a family as an interactive system sharing stories and emotions. It is especially important in pediatric psychology practice, as was noted by structural family therapist Salvadore Minuchin, who described family patterns and family interventions while treating families with children with chronic illnesses. In one of his works, he wrote, “that in some families, at least, stress between parents can be measured in the bloodstream of their observing child” [39]. Following this circular and perpetual process of mutual affecting is a great challenge especially in the situation of a sudden change, for example, in a form of hospitalization. A parent’s disorganization, coupled with emotional instability, can have a profound effect on her/his ability to support and meet the needs of a sick child, significantly lowering the child’s quality of life [40]. In a hospital environment, a parent’s higher anxiety score is associated with lower optimism scores and a higher level of illness-related uncertainty, which might influence the process of a child’s health outcome, health monitoring, and in the end, treatment decisions. Parents under severe stress will probably be the parents requiring special attention and one with whom cooperation may potentially be more difficult. That is the reason why sometimes family caregivers are called “secondary patients” [41] who need additional protection and guidance as a form of support from medical staff, and that is the reason why diagnosing parental stress should be essential. By adjusting the way of communicating and cooperating with the emotional/temperamental needs of parents, medical staff can contribute to a significant reduction in the intensity of stress. Parental participation, as indicated by Lam et al., (2006) should provide personalized care as much as possible and the ability to meet the emotional needs of both children and parents [36]. Power and Franck (2008) recommend that parents should be informed, supported, and involved in the day-to-day care plan, in addition to being involved in the decision-making process [2]. These factors reduce the level of experienced stress and anxiety among parents related to the lack of information about the disease, medical procedures used, ignorance of hospital regulations, and fear of asking questions [42]. Continuing this thought, Barr (2011) [9] indicates that reducing anxiety has a positive effect on the well-being of parents and children, and consequently on the recovery process. The author also points out that relieving parents’ stress increases their satisfaction with the hospitalization. The correct flow of information between medical staff and parents of the hospitalized child, contributing to the reduction in anxiety and experienced stress, also encourages better cooperation and increases understanding of the actions taken by medical services [37].

In empirical studies, parental stress was also found to be significantly related to parental satisfaction with hospitalization. Improvement in satisfaction was also observed as a result of entrusting mothers with partial responsibility for childcare [36]. In addition, parents are happier when they have the opportunity to ask questions, when professionals take their concerns into account, provide useful information, and engage in a supportive manner [37]. Positive collaboration with healthcare professionals, including factors such as being serious about the parents’ concerns, treating them as partners in decision making, and explaining procedures, significantly reduced uncertainty, leading to increased satisfaction and reduced stress levels [38]. Therefore, good relations with professionals may contribute to the fact that the child’s stay in the hospital will be less stressful, for both the family and the child, and thus will have a positive impact on communication, but also on the recovery process of the child [43].

## 5. Conclusions

1. Taking into account individual factors such as temperament and stress reactions is necessary for the process of diagnosing the family system in which the child is hospitalized;
2. Although it is not a predictor of parental stress, social support obtained by relatives and staff requires further research;
3. Regardless of the child’s age, parental dissatisfaction is the moderator of stress. At the same time, in the parents of younger children (3–6 years old), the child’s emotionality, and in older children (7–14 years old), the child’s health are the predictors of parental stress;
4. High levels of stress increase the temperamentally difficult characteristics of the child and the parent; therefore, lowering the anxiety of the parent and taking care of the emotional well-being of the child is advisable.

## Figures and Tables

**Table 1:** Characteristic of the parent group.

	Younger Group of Children (*n* = 108)	Older Group of Children (*n* = 95)	Total (*N* = 203)
Gender, % (*n*)			
emale	86.1 (93)	88.4 (84)	87.2 (177)
Male	13.9 (15)	11.6 (11)	12.8 (26)
Age, M ± SD	34.45 ± 5.12	37.00 ± 5.59	35.65 ± 5.48
Range of age	23–48	27–59	23–59
Level of education, % (*n*)			
Primary	5.6 (6)	5.3 (5)	5.4 (11)
Vocational	12.0 (13)	8.4 (8)	10.3 (21)
Secondary	28.7 (31)	26.3 (25)	27.6 (56)
Higher	53.7 (58)	60.0 (57)	56.7 (115)
Employment, % (*n*)			
Employment contract	65.7 (71)	71.6 (68)	68.5 (139)
Own business	9.3 (10)	10.5 (10)	9.9 (20)
Unemployment	19.4 (21)	13.7 (13)	16.7 (34)
Other sources of employment	4.6 (5)	4.2 (4)	4.4 (9)
Sickness pension	0.9 (1)	0.0 (0)	0.5 (1)
Income level, % (*n*)			
Low	1.9 (2)	4.2 (4)	3.0 (6)
Average	36.1 (39)	32.6 (31)	34.5 (70)
Good	44.4 (48)	51.6 (49)	47.8 (97)
Very good	17.6 (19)	11.6 (11)	14.8 (30)

**Table 2:** Characteristic of children group.

	Younger Group of Children (*n* = 108)	Older Group of Children (*n* = 95)	Total (*N* = 203)
Gender, % (*n*)			
emale	50.9 (55)	56.8 (54)	53.7 (109)
Male	49.1 (53)	43.2 (41)	46.3 (94)
Age, M ± SD	4.93 ± 0.88	8.82 ± 1.59	6.75 ± 2.32
Range of age	3–6	7–14	3–14
Length of hospitalization (days), M ± SD	3.28 ± 1.81	3.63 ± 3.55	3.44 ± 2.77
Range of length of hospitalization (days)	1–9	1–23	1–23
Care in the hospital, % (*n*)			
Both parents	21.3 (23)	18.9 (18)	20.2
Mainly mother	73.1 (79)	70.5 (67)	71.9
Mainly father	5.6 (6)	8.4 (8)	6.9
Another person	0.0 (0)	2.1 (2)	1.0

**Table 3:** Correlations between parental stress and other variables.

	Child Health	Disruption of Life	Family Support	Hospital Staff Support
Stress	−0.164 *	0.88	−0.195 **	0.119

* *p* < 0.05, ** *p* < 0.01.

**Table 4:** Correlations between temperamental traits and parental stress.

	EASDED	EASDEF	EASDEA	EASDA	EASDS	EASCE	EASCA	EASCT	EASCN
Stress	0.550 ***	0.221 **	0.384 ***	−0.049	−0.156 *	0.280 ***	0.024	0.056	0.119

Note. EASDED = parental emotionality—distress; EASDEF = parental emotionality—fear; EASDEA = parental emotionality—anger; EASDA = parental activity; EASDS = parental sociability; EASCE = child’s emotionality; EASCA = child’s activity; EASCS = child’s sociability; EASCS = child’s shyness. * *p* < 0.05, ** *p* < 0.01, *** *p* < 0.001.

**Table 5:** Regression analysis with interaction effects for parental stress.

Variable	*B*	95% CI for B		*SE B*	β	*R* ^2^	∆*R*^2^
		*LL*	*UL*				
Step 1						0.395	0.343 ***
Constant	7.441	−4.106	18.989	5.848			
Age of the child	−0.354	−1.276	0.567	0.467	−0.048		
Child health	−0.244	−0.747	0.259	0.255	−0.063		
Disruption of life	−0.049	−0.447	0.349	0.202	−0.017		
Family support	−0.501 *	−0.908	−0.095	0.206	−0.162 *		
Hospital support	−0.063	−0.597	0.472	0.271	−0.016		
EASDED	1.202 ***	0.830	10.573	0.188	0.543 ***		
EASDEF	0.068	−0.218	0.355	0.145	0.031		
EASDEA	0.050	−0.334	0.434	0.194	0.021		
EASDA	−0.352 *	−0.703	−0.002	0.178	−0.141 *		
EASDS	0.092	−0.308	0.492	0.203	0.032		
EASCE	0.201	−0.088	0.491	0.146	0.101		
EASCA	0.175	−0.085	0.434	0.131	0.100		
EASCS	0.010	−0.306	0.327	0.160	0.005		
EASCS	−0.016	−0.280	0.248	0.134	−0.009		
Step 2						0.464	0.368 ***
Constant	2.176	−10.291	14.644	6.310			
Age of the child	−0.311	−1.221	0.599	0.461	−0.042		
Child health	−0.244	−0.762	0.275	0.262	−0.063		
Disruption of life	−0.056	−0.454	0.342	0.201	−0.019		
Family support	−0.428 *	−0.832	−0.023	0.205	−0.138 *		
Hospital support	0.012	−0.536	0.561	0.277	0.003		
EASDED	1.240 ***	0.839	1.641	0.203	0.560 ***		
EASDEF	0.066	−0.288	0.421	0.179	0.030		
EASDEA	−0.022	−0.409	0.366	0.196	−0.009		
EASDA	−0.306	−0.686	0.074	0.192	−0.123		
EASDS	0.046	−0.361	0.452	0.206	0.016		
EASCE	0.274	−0.037	0.585	0.157	0.137		
EASCA	0.135	−0.125	0.395	0.132	0.078		
EASCS	0.181	−0.165	0.528	0.175	0.088		
EASCS	0.084	−0.197	0.366	0.143	0.051		
Child health × age	0.554 *	0.035	1.072	0.262	0.143 *		
Disruption of life × age	−0.114	−0.512	0.284	0.201	−0.039		
Family support × age	−0.076	−0.481	0.328	0.205	−0.025		
Hospital support × age	0.429	−0.119	0.977	0.277	0.110		
EASDED × age	−0.041	−0.442	0.361	0.203	−0.018		
EASDEF × age	0.063	−0.292	0.417	0.179	0.028		
EASDEA × age	−0.347	−0.735	0.041	0.196	−0.148		
EASDA × age	−0.077	−0.457	0.303	0.192	−0.031		
EASDS × age	−0.061	−0.467	0.346	0.206	−0.021		
EASCE × age	0.121	−0.190	0.432	0.157	0.060		
EASCA × age	0.036	−0.224	0.296	0.132	0.021		
EASCS × age	−0.365 *	−0.711	−0.018	0.175	−0.176 *		
EASCS × age	−0.111	−0.393	0.170	0.143	−0.067		

Note. EASDED = parental emotionality—distress; EASDEF = parental emotionality—fear; EASDEA = parental emotionality—anger; EASDA = parental activity; EASDS = parental sociability; EASCE = child’s emotionality; EASCA = child’s activity; EASCS = child’s sociability; EASCS = child’s shyness; variable × age = interaction between variables. * *p* < 0.05, *** *p* < 0.001.

**Table 6:** Stress predictors in the group of parents of younger children.

Variable	*B*	95% CI for B		SE B	β	R^2^	∆R^2^
		*LL*	*UL*				
Step 3						0.410	0.314 ***
Constant	7.160	−8.080	22.400	7.658			
Child health	0.310	−0.376	0.996	0.345	0.088		
Disruption of life	−0.171	−0.719	0.378	0.275	−0.063		
Family support	−0.504	−1.046	0.037	0.272	−0.174		
Hospital support	0.441	−0.299	1.182	0.372	0.119		
EASDED	1.199 ***	0.677	1.722	0.263	0.623 ***		
EASDEF	0.129	−0.483	0.740	0.307	0.045		
EASDEA	−0.369	−0.920	0.183	0.277	−0.162		
EASDA	−0.383	−0.825	0.059	0.222	−0.178		
EASDS	−0.015	−0.577	0.547	0.282	−0.006		
EASCE	0.395 *	0.021	0.769	0.188	0.239 *		
EASCA	0.171	−0.175	0.518	0.174	0.106		
EASCS	−0.183	−0.562	0.195	0.190	−0.108		
EASCS	−0.027	−0.374	0.320	0.174	−0.019		

Note. EASDED = parental emotionality—distress; EASDEF = parental emotionality—fear; EASDEA = parental emotionality—anger; EASDA = parental activity; EASDS = parental sociability; EASCE = child’s emotionality; EASCA = child’s activity; EASCS = child’s sociability; EASCS = child’s shyness; variable × age = interaction between variables. * *p* < 0.05, *** *p* < 0.001.

**Table 7:** Stress predictors in the group of parents of older children.

Variable	*B*	95% CI for B		SE B	β	R^2^	∆R^2^
		*LL*	*UL*				
Step 3						0.508	0.417 ***
Constant	−2.807	−23.157	17.543	10.203			
Child health	−0.797 *	−1.594	−0.001	0.400	−0.188 *		
Disruption of life	0.058	−0.530	0.647	0.295	0.018		
Family support	−0.351	−0.966	0.263	0.308	−0.107		
Hospital support	−0.416	−1.243	0.410	0.415	−0.102		
EASDED	1.280 ***	0.656	1.905	0.313	0.493 ***		
EASDEF	0.004	−0.336	0.343	0.170	0.002		
EASDEA	0.325	−0.228	0.879	0.278	0.135		
EASDA	−0.229	−0.869	0.411	0.321	−0.078		
EASDS	0.106	−0.493	0.706	0.301	0.034		
EASCE	0.152	−0.360	0.665	0.257	0.059		
EASCA	0.099	−0.298	0.496	0.199	0.053		
EASCS	0.546	−0.056	1.148	0.302	0.206		
EASCS	0.196	−0.262	0.653	0.229	0.099		

Note. EASDED = parental emotionality—distress; EASDEF = parental emotionality—fear; EASDEA = parental emotionality—anger; EASDA = parental activity; EASDS = parental sociability; EASCE = child’s emotionality; EASCA = child’s activity; EASCS = child’s sociability; EASCS = child’s shyness; variable × age = interaction between variables. * *p* < 0.05, *** *p* < 0.001.

## Data Availability

We do not report any data.

## References

[1] Tsironi S., Koulierakis G. (2018). Factors associated with parents’ levels of stress in pediatric wards. J. Child Health Care.

[2] Power N., Franck L. (2008). Parent participation in the care of hospitalized children: A systematic review. J. Adv. Nurs..

[3] Franck L.S., Wray J., Gay C., Dearmun A.K., Lee K., Cooper B.A. (2015). Predictors of parent post-traumatic stress symptoms after child hospitalization on general pediatric wards: A prospective cohort study. Int. J. Nurs. Stud..

[4] Dunn M.J., Rodriguez E.M., Barnwell A.S., Grossenbacher J.C., Vannatta K., Gerhardt C.A., Compas B.E. (2012). Posttraumatic stress symptoms in parents of children with cancer within six months of diagnosis. Health Psychol..

[5] Hoekstra-Weebers J.E., Wijnberg-Williams B.J., Jaspers J.P., Kamps W.A., van de Wiel H.B. (2012). Coping and its effect on psychological distress of parents of pediatric cancer patients: A longitudinal prospective study. Psycho-Oncology.

[6] Helle N., Barkmann C., Ehrhardt S., von der Wense A., Nestoriuc Y., Bindt C. (2016). Postpartum anxiety and adjustment disorders in parents of infants with very low birth weight: Cross-sectional results from a controlled multicentre cohort study. J. Affect. Disord..

[7] Colville G., Darkins J., Hesketh J., Bennett V., Alcock J., Noyes J. (2009). The impact on parents of a child’s admission to intensive care: Integration of qualitative findings from a cross-sectional study. Intensive Crit. Care Nurs..

[8] Helfricht S., Latal B., Fischer J.E., Tomaske M., Landolt M.A. (2008). Surgery-related posttraumatic stress disorder in parents of children undergoing cardiopulmonary bypass surgery: A prospective cohort study. Pediatr. Crit. Care Med..

[9] Barr P. (2011). Posttraumatic growth in parents of infants hospitalized in a neonatal intensive care unit. J. Loss Trauma.

[10] Murray B.L., Kenardy J.A., Spence S.H. (2008). Brief report: Children’s responses to trauma-and nontrauma-related hospital admission: A comparison study. J. Pediatr. Psychol..

[11] Bonn M. (1994). The effects of hospitalisation on children: A review. Curationis.

[12] Tremolada M., Bonichini S., Basso G., Pillon M., Columbus A.M. (2015). Develompment delays and temperament in children with leukemia after the first year of therapy. Advances in Psychology Research.

[13] Lumley M.A., Melamed B.G., Abeles L.A. (1993). Predicting children’s presurgical anxiety and subsequent behavior changes. J. Pediatr. Psychol..

[14] Melnyk B.M., Feinstein N.F. (2001). Mediating functions of maternal anxiety and participation in care on young children’s posthospital adjustment. Res. Nurs. Health.

[15] Kirkby R.J., Whelan T.A. (1996). The effects of hospitalisation and medical procedures on children and their families. J. Fam. Stud..

[16] Rettew D.C., Stanger C., McKee L., Doyle A., Hudziak J.J. (2006). Interactions between child and parent temperament and child behavior problems. Compr. Psychiatry.

[17] Chess S., Thomas A., Strelau J., Angleitner A. (1991). Temperament and the concept of goodness of fit. Explorations in Temperament.

[18] Cohen S., Kamarck T., Mermelstein R. (1983). A Global Measure of Perceived Stress. J. Health Soc. Behav..

[19] Juczyński Z., Ogińska-Bulik N. (2012). Narzędzia Pomiaru Stresu i Radzenia Sobie ze Stresem.

[20] Buss A.H., Plomin R. (1975). A Temperament Theory of Personality Development.

[21] Oniszczenko W. (1997). Kwestionariusz Temperamentu EAS.

[22] Bedyńska S., Książek M. (2012). Statystyczny Drogowskaz: Praktyczny Przewodnik Wykorzystania Modeli Regresji Oraz Równań Strukturalnych.

[23] Erikson E.H. (2000). Dzieciństwo i Społeczeństwo.

[24] Gurtovenko K., Fladeboe K.M., Galtieri L.R., King K., Friedman D., Compas B., Breiger D., Lengua L., Keim M., Kawamura J. (2021). Stress and psychological adjustment in caregivers of children with cancer. Health Psychol..

[25] Toledano-Toledano F., Luna D. (2020). The psychosocial profile of family caregivers of children with chronic diseases: A cross-sectional study. BioPsychoSocial Med..

[26] Commodari E. (2010). Children staying in hospital: A research on psychological stress of caregivers. Ital. J. Pediatr..

[27] Tiedeman M.E. (1997). Anxiety responses of parents during and after the hospitalization of their 5-to 11-year-old children. J. Pediatric Nurs..

[28] Hallström I., Runesson I., Elander G. (2002). Observed parental needs during their child’s hospitalization. J. Pediatr. Nurs..

[29] Krywda-Rybska D., Zdun-Ryżewska A., Zach E. (2012). Psychological stress and its causes for caregivers of children hospitalized for short time. Pediatr. Med. Rodz..

[30] Knox J.E., Hayes V.E. (1983). Hospitalization of a Chronically Ill Child: A Stressful Time for Parents. Issues Compr. Pediatr. Nurs..

[31] Hilliard M.E., Monaghan M., Cogen F.R., Streisand R. (2011). Parent stress and child behaviour among young children with type 1 diabetes. Child Care Health Dev..

[32] Mazurek Melnyk B., Fischbeck Feinstein N., Zendi M., Leigh S. (2001). Coping in parents of children who are chronically ill: Strategies for assessment and intervention. Pediatr. Nurs..

[33] Bętkowska-Korpała B., Gierowski J.K. (2007). Psychologia Lekarska w Leczeniu Chorych Somatycznie.

[34] Loewenstein K., Barroso J., Phillips S. (2019). The Experiences of Parents in the Neonatal Intensive Care Unit. J. Perinat. Neonatal Nurs..

[35] Kazak A.E., Simms S., Rourke M.T. (2002). Family systems practice in pediatric psychology. J. Pediatr. Psychol..

[36] Lam L.W., Chang A.M., Morrissey J. (2006). Parents’ experiences of participation in the care of hospitalised children: A qualitative study. Int. J. Nurs. Stud..

[37] Osborne L.A., Reed P. (2008). Parents’ perceptions of communication with professionals during the diagnosis of autism. Autism.

[38] Moh T.A., Magiati I. (2012). Factors associated with parental stress and satisfaction during the process of diagnosis of children with Autism Spectrum Disorders. Res. Autism Spectr. Disord..

[39] Minuchin S., Fishman H.C. (2002). Family Therapy Techniques.

[40] Almogbel Y.S. (2016). Parenting Stress and Child’s Quality of Life on Parents’ Expectation/Satisfaction and Medication Adherence with Asthmatic Child’s Therapy. Ph.D. Thesis.

[41] Reinhard S.C., Given B., Petlick N.H., Bemis A., Hughes R.G. (2008). Supporting Family Caregivers in Providing Care. Patient Safety and Quality: An Evidence-Based Handbook for Nurses.

[42] Tehrani T.H., Haghighi M., Bazmamoun H. (2012). Effects of stress on mothers of hospitalized children in a hospital in Iran. Iran. J. Child Neurol..

[43] Tsironi S., Koulierakis G. (2019). Factors affecting parents’ satisfaction with pediatric wards. Jpn. J. Nurs. Sci..

